# Effect of Storage on the Physicochemical and Flavour Attributes of Two Cultivars of Strawberry Cultivated in Northern India

**DOI:** 10.1155/2014/794926

**Published:** 2014-01-23

**Authors:** Rachna Mishra, Abhijit Kar

**Affiliations:** Division of Post Harvest Technology, Indian Agricultural Research Institute, Pusa Campus, New Delhi 110 012, India

## Abstract

An attempt was made to understand the changes in physicochemical quality (total soluble solids, titratable acidity, ascorbic acid, total sugars, total phenolics, anthocyanin content, and cupric reducing antioxidant capacity assay) and total volatile/aroma compounds of two cultivars of strawberry (Camarosa and Chandler) during storage at 5°C for 9 days at an interval of three days. Observations indicated a significant quantitative difference both in the physicochemical and in total volatile content among the cultivars indicating the importance of cultivar for determining the postharvest quality and shelf life. At the end of 9 days of storage significant changes in the physicochemical and total volatile/aroma compounds were observed. Total antioxidants and total phenols were found to increase significantly, whereas total soluble solids and total sugars decreased with the advent of storage period for both cultivars. Total anthocyanin contents however remained almost constant throughout the storage period. Titratable acidity in Camarosa reduced with the increase in the storage period whereas it remained almost constant in Chandler. Ascorbic acid increased in Camarosa whereas the same decreased significantly in Chandler. Significantly higher contents of esters and terpenoids in Camarosa indicated a better retention of the typical fruits flavour of strawberry compared to that of Chandler.

## 1. Introduction

Intake of fresh fruits and vegetables helps in preventing a number of chronic diseases like cancer and cardiovascular diseases [1]. Increased consumer demand for high quality fruit having attractive appearance, high nutritional value, and good taste combined with the reported health benefits from biologically active components like antioxidants and phenolics [2] has enabled enhanced attention among the researchers for the retention of the quality (both physicochemical and flavour) of the fresh fruits.

Strawberry (*Fragaria x ananassa *Duch.) is one of the most popular, nutritious fruits among all the berries and is cultivated in almost all countries of the world. It has a delicious taste and unique flavor widely accepted and consumed in fresh or processed form. Aroma in strawberry mainly constitutes a complex mixture of esters, aldehydes, alcohols, and sulfur compounds. Among the hundreds of volatiles identified in strawberry, esters are known to be the most important contributors to its typical fruity flavor. Differences in strawberry aroma compounds among cultivars have been reported by several authors [3, 4]. In addition to its attractive flavour strawberry has an extremely attractive bright red appearance because of anthocyanins. The anthocyanin found predominantly constitutes pelargonidin 3-glucoside. The other minor ones include cyanidin 3-glucoside and pelargonidin 3-rutinoside [5]. In addition to its flavouring compounds and anthocyanins, strawberry is also found to be rich in antioxidant [6, 7], flavonols [6], phenolic compounds [8], and ascorbic acid [9].

Strawberries are highly perishable having a very limited shelf life [10]. Low temperature storage helps in extending the shelf life to some extent [11]. In fact several studies have been aimed in the past toward the extension of the shelf life of strawberry [3, 5]. Most of the biochemical constituents such as anthocyanins, vitamin C, antioxidant, total phenolics, and flavour are greatly affected by the cultivar, stage of harvest, and the postharvest storage conditions.

Strawberry is an important fruit with respect to its nutritional content as well as its flavour. Flavor of strawberry is considered to be an important aspect of its quality. Although difficult to define, qualify, and quantify, this elusive and complex trait is important to consumers and deserves adequate attention. Flavor quality of strawberry is an important factor in an increasingly competitive global market. Flavor maintenance becomes a challenge to maintain as shelf life and marketing distances increase due to new storage, handling, and transport technologies.

Flavor and aroma are perhaps the most elusive and subjective quality traits. Flavor mainly constitutes a combination of sweetness and sourness, whereas aroma represents the mixture of sugars, acids, and volatiles. Volatile biosynthesis and its contribution to fruit eating quality are very complex, and are influenced by many factors, such as cultivar, harvest maturity, postharvest handling, and storage [12, 13].

Measurement of aroma compounds is difficult and time consuming. The disadvantage of classical flavor isolation procedures of steam distillation and/or solvent extraction is that it could qualitatively and quantitatively modify the flavor profile of a sample. Static headspace methods have replaced the traditional technique as it is said to more closely reflect the true flavor profile, but compounds which are present at low levels may not be detected at all. Therefore, solid phase microextraction (SPME) which is a rapid sampling technique, where volatiles interact with a fiber-coated probe inserted into the sample headspace, has been developed and widely adopted.

Hence, an attempt was made to study a quantitative analysis of physicochemical parameters (total soluble solids, titratable acidity, ascorbic acid, total sugars, total phenolics, anthocyanin content, and cupric reducing antioxidant capacity assay) and total volatile/aroma compounds of two predominantly grown cultivars of strawberries (Camarosa and Chandler) in order to understand the qualitative changes during its storage at 5°C. These two varieties were selected since they are predominately cultivated in India.

## 2. Materials and Methods

### 2.1. Material

About 30 kg of strawberries fruits of each of the varieties (Cv. Camarosa and Chandler) of uniform size and colour were freshly harvested from a private farmer's field, washed thoroughly to remove any extraneous material, surface-dried using blotting paper, divided into three lots per variety, and were stored at 5 ± 1°C and 85 ± 5% relative humidity. Fruits were evaluated for physicochemical and volatile content at the beginning and subsequently at three-day intervals up to the 9th day. All analyses were performed in triplicate and results were expressed as fresh weight (f.w.) basis.

### 2.2. Total Soluble Solids (TSS) and Titratable Acidity (TA) Determinations

TSS was analyzed with the help of a hand held digital refractometer whereas TA was determined by titration method using 0.1 N NaOH and phenolphthalein as an indicator [14].

### 2.3. Ascorbic Acid (AA) and Total Sugar Determination

2 g of crushed sample was extracted with metaphosphoric acid (3%) and the total AA was determined by titrating a known weight of sample against 2,6-dichlorophenol-indophenol dye [14]. Result was expressed in mg/100 g of fresh fruit. Total sugar was determined by using Lane and Eynon method [14].

### 2.4. Determination of Total Phenols

Total phenols were quantified using 1 g homogenate (pulp homogenized in a blender) using the method described by Singleton et al. [15]. Total phenol was extracted in methanol (80%) and left for 2 hrs. The extract was centrifuged at 10,000 rpm for 15 min. About 0.1 mL of the supernatant was mixed with 2 mL of saturated sodium carbonate, 0.5 mL of Folin-Ciocalteu reagent, and 2.9 mL of distilled water and incubated at 27°C for 1 hr and the absorbance read at 765 nm using a spectrophotometer. Total phenolic content was expressed as gallic acid equivalents in mg/100 g fresh weight.

### 2.5. Total Anthocyanins Content

Total anthocyanin contents were determined using pH differential spectrophotometric (absorbance at 520 and 700 nm) method [16] and expressed as milligram pelargonidin-3-glucoside (molar extinction coefficient of 22,400 and molecular weight of 433.2 g/mol) per 100 g fresh weight.

### 2.6. Cupric Reducing Antioxidant Capacity (CUPRAC) Assay

CUPRAC assay was done by the method developed by Apak et al. [17]. To 100 *μ*L of sample aliquot, 1 mL each of copper (II) chloride solution (10^−2 ^M), neocuproine solution (7.5 × 10^−3 ^M), and ammonium acetate buffer solution (pH 7) solution were mixed. The tubes were stoppered and, after 1 h, absorbance at 450 nm was recorded against a reagent blank. Results were expressed as *μ*mol TE/g.

### 2.7. Analysis of Aroma Compounds/Volatiles

Headspace sampling method was adopted using a 100 *μ*m fused-silica fibre coated with polydimethylsiloxane (PDMS) (Supelco, Bellefonte, PA, USA). 5 g of the homogenized fruit pulp was taken in a 20 mL vial and subjected to GC-MS analysis. Sample was first equilibrated for 10 min at 38°C and maintained at 38°C throughout the 30 min assay. After 30 min of extraction the SPME syringe was introduced into the injector port at a temperature of 240°C using splitless injection for further analysis. Volatile compounds were analysed using a Varian 450-GC/240-MS apparatus equipped with a VF-5 ms (30 m × 0.25 mm × 0.25 *μ*m) fused-silica capillary column. Helium (1 mL/min) was used as a carrier gas. The oven operated at the following ramp rates: 40°C–120°C at 6°C/min; 70°C–170°C at 4°C/min; and 180°C–240°C at 6°C/min. Thermal desorption was subsequently allowed for 15 min. The transfer line and ion source temperatures were maintained at 280°C and 180°C, respectively. The energy of electron was 70 eV and ion mass/charge ratio 20–450 m/z under full scan mode. The components were identified by comparison of mass spectra and retention time data with Wiley and NIST library and authentic standards. Quantification was achieved by using 3-chlorophenol as an internal standard.

### 2.8. Statistical Analysis

Experiments were performed according to completely randomized design having three replications. Two factor Analysis of Variance (ANOVA) with cultivar and time as factors was performed using PROC GLM of SAS to understand the effect of time period and variety on the fruit quality (TSS, Titratable Acidity, Ascorbic Acid, Total Sugar, Total Phenolics, Total Anthocyanin, Antioxidant Capacity, and Volatiles).

## 3. Results and Discussion

### 3.1. TSS, TA, AA, and Total Sugar

**Figure 1:**
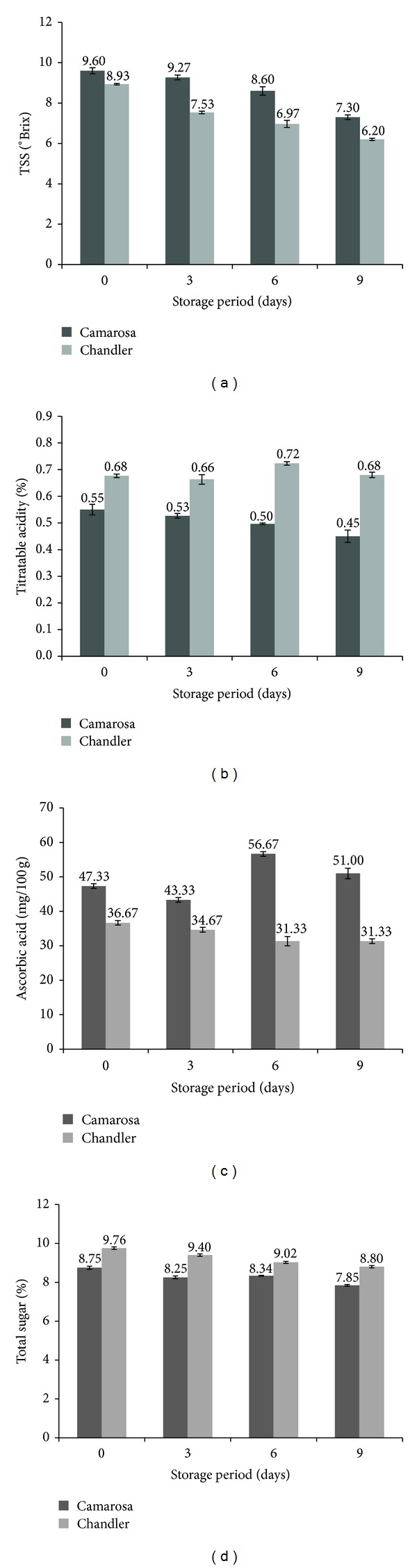
Changes in physicochemical parameters of strawberries during storage at 5°C (a) TSS, (b) titratable acidity, (c) ascorbic acid, and (d) total sugar.

TSS for both cultivars decreased with the increase in the storage period (Figure 1(a)). Both varieties were found to be significantly different in their TSS contents. For both varieties, storage period was found to have a significant effect on the TSS content. The decrease in the TSS content after nine days of storage was more for Chandler (about 30%) in comparison to Camarosa (about 24%). The significant loss suggests decrease in total sugars and may be attributed to the enhanced respiration rate of the fruits during storage. Similar results have been reported by Gil et al. [5] and Pelayo et al. [3].

TA has been expressed in terms of percentage citric acid since citric acid constitutes a major chunk of the acids in strawberry [3, 18]. TA in case of Camarosa decreased with the advent of the storage period (Figure 1(b)). Pelayo et al. [3] also reported similar results in three different strawberry cultivars. However, it remained almost constant even at the end of 9th day of storage in case of Chandler. TA of both cultivars was found to be significantly different from each other. Chandler variety showed a significantly higher TA content compared to Camarosa throughout the storage period.

Ascorbic acid, a predominant form of vitamin C, is known to be highly unstable. Temperature is reported to have a significant effect on the ascorbic acid retention. The AA content reduced with the increase in the storage period for Chandler which could be attributed to the activity of ascorbate oxidase which promotes ascorbic acid to dehydroascorbic acid [19]. The decrease was about 15% (Figure 1(c)). On the other hand, the AA content fluctuated quite a bit with the storage period in case of Camarosa. However, the total AA content increased at the end of 9 days of storage indicating synthesis of ascorbic acid during storage as reported by Cordenunsi et al. [20].

High sugar and high acid content are the known precursors of good strawberry flavor. Total sugar content also followed a trend similar to that of TSS. However, at the end of the nine-day storage period, both cultivars have almost similar total sugar loss (Figure 1(d)). In this case also ANOVA indicated a significant effect of both storage period and variety. The loss in sugars can be mostly explained by the hydrolysis of sucrose and the utilization of the corresponding reducing sugars in the fruit respiration since strawberry does not have much starch to support total sugar synthesis after harvest. Castro et al. [21] also reported loss of total sugar in strawberries during storage.

### 3.2. Total Anthocyanin, Total Phenols, and CUPRAC Assay

**Figure 2:**
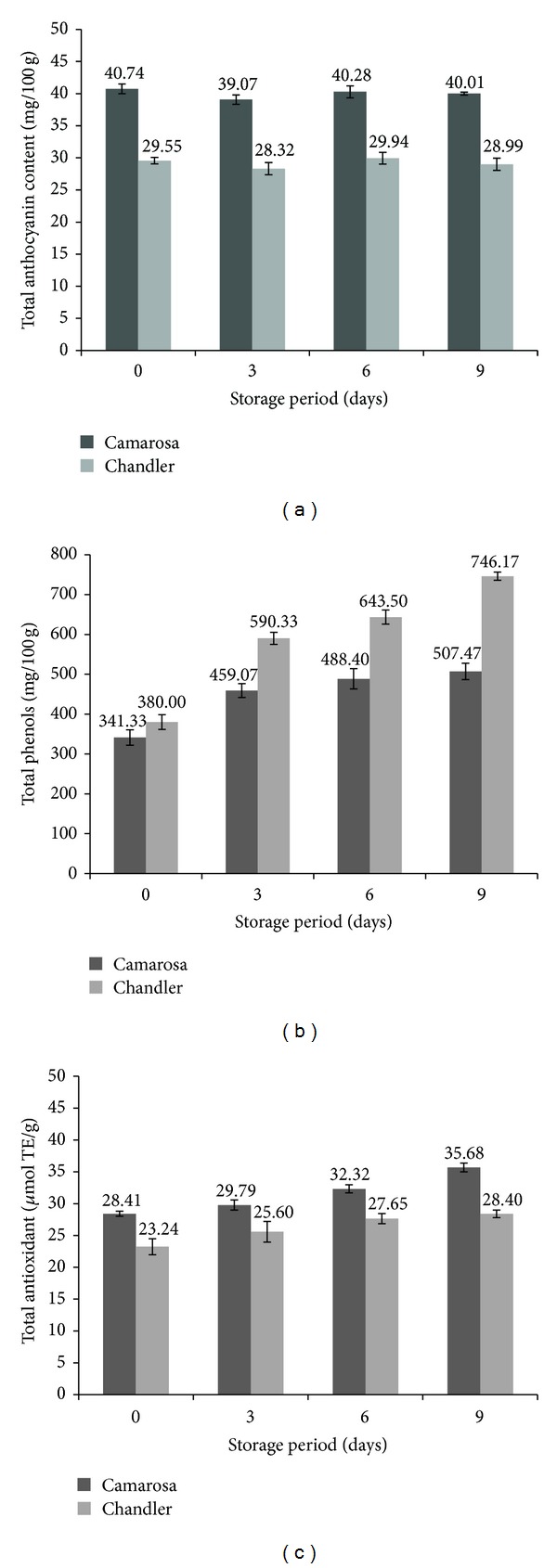
Changes in (a) total anthocyanin content, (b) total phenols, and (c) Total antioxidants of strawberry (Camarosa and Chandler) during storage at 5°C.

Pelargonidin-3-glucoside is the most prevalent anthocyanin pigment which is responsible for red color of strawberry. Camarosa and Chandler were found to have significantly different total anthocyanin contents (Figure 2(a)) which can be mainly attributed to the varietal characteristics. ANOVA indicated a nonsignificant effect of storage period on the total anthocyanin content for both cultivars which indicates that the colour of fruits of both varieties was retained at the end of 9 days of storage. Kalt and McDonald [22] have reported an increase in the anthocyanin content of strawberry during storage whereas Ayala-Zavala et al. [23] reported a decrease in anthocyanin content when the strawberries were stored at 5°C. The biosynthetic pathway for the anthocyanin is known to be uninhibited by storage at low temperatures [22]. However, in the varieties under consideration in this study the pathway seems to have been sufficiently inhibited so as not to inflict any changes in the total anthocyanin content even after 9 days of storage.

The initial total phenol content of both varieties was slightly different from each other but was not found to be significantly different from each other (Figure 2(b)). However, the difference becomes statistically significant all the subsequent period of storage under consideration. A remarkable increase in the total phenols was observed in both cultivars which may be attributed to the hydrolysis of the tannins which are reported to be dominant in strawberries [24].

Antioxidant activity of fruit can be estimated using ferric reducing antioxidant power (FRAP), oxygen radical absorbance capacity (ORAC), or cupric reducing antioxidant capacity (CUPRAC) assay. CUPRAC assay for the estimation of antioxidant capacity was adopted for this study because of its distinctive documented advantages like simplicity, clarity of endpoint, readily available instrumentation, good intra- and interassay reproducibility, and high throughout for routine analysis by Prior et al. [25]. Variety as well as storage period had a significant effect on the antioxidant activity exhibited by CUPRAC assay. The antioxidant activity increased with the increase in the storage period for both varieties. However, the increase was higher in Camarosa for each difference in time periods than that of Chandler (Figure 2(c)). The results are in agreement with the ORAC values reported by Ayala-Zavala et al. [23].

Kalt and McDonald [22] also reported changes in phenolic composition, anthocyanin, and antioxidant capacities in strawberry, raspberry, and highbush and lowbush blueberries during postharvest storage treatments. They also reported correlation of antioxidant capacity with phenolic and anthocyanin content.

### 3.3. Aroma Compound

Typical aroma of strawberries comes from not only one or a few impact aroma compounds, but also from numerous volatiles present at certain concentrations and in a particular balance among them. Thus, strawberry aroma is a result of a combined perception of many aromatic constituents [26].

The aroma profile did not vary qualitatively but only quantitatively among genotypes in their aroma profile (Table 1). Both produced the same major aroma compounds (esters, terpenoids, and furanones) with the exceptions of mesifurane which was not found in Chandler and nerolidol which was found in significantly higher quantity in Camarosa compared to Chandler.

Esters are one of the most important volatile compounds in fruit flavour. Fruity, green grass and other flavour notes of strawberries are emanated by a complex mixture of esters [26]. Esters accounted for the majority of the aroma compounds in both cultivars. Prominent among the esters were methyl hexanoate, ethyl hexanoate, hexyl hexanoate, propyl hexanoate, n-octyl butyrate, and n-octyl acetate. The total esters content of Camarosa reduced with the advent of the storage period, whereas that of Chandler increased significantly with the increase in the storage period. The increase in Chandler is mainly due to the enhanced production of methyl octanoate which can be attributed to the activity of fermentative and volatile biosynthesis related enzymes and isoenzyme in response to senescence and stress conditions [3].

The terpenoid profile of both varieties was dominated by nerolidol and linalool which are considered to be an important contributor to the fresh aroma of strawberry. Nerolidol was found to be significantly higher in Camarosa than in Chandler. However, the same significantly decreased with the increase in the storage period.

Many short-chain alcohols and acids have been found in strawberries [27]; however, since most of short-chain alcohols and acids have very high sensory thresholds, they contribute very little to the overall aroma of strawberry and thus their concentrations were not quantified. Concentrations of alcohols were found to be higher in Camarosa compared to those of Chandler. Hexanal, the only aldehyde identified and associated with the green and cut grass odour characteristics in fruits, was found in higher concentration (almost 50% more) in Camarosa compared to that in Chandler.

Furanones are also the main contributors to the aroma of fresh strawberries other than esters. 2,5-Dimethyl-4-hydroxy-3(2H)-furanone (furaneol) and mesifurane were the two furanones which were found in Camarosa. Both furaneol and mesifurane have strong, sweet, and pleasant odors. Furaneol imparts caramel burnt sugar notes at high concentrations and becomes fruity at lower concentrations [28]. Chandler, however, did not have any detectable mesifuran. Mesifurane is described as having a more sherry-like aroma [29]. Recently, Larsen and Poll [30] found that a mixture of furaneol and ethyl butanoate presented a strawberry-like odor. Total furanone content was retained over the period of storage in Camarosa while in case of Chandler it was totally lost by the end of the 9 days of storage.

Among other compounds, *α*-farnesene and dodecalactone were found in Camarosa. However, dodecalactone was found only in Camarosa. *α*-Farnesene was found to increase with the storage period for both varieties, whereas dodecalactone remained constant over the entire storage period.

## 4. Conclusions

The present study revealed that storage of strawberry has a significant effect on the physicochemical parameters and aroma volatiles of strawberry. Total antioxidants and total phenols were found to increase significantly for both varieties with the increase in the storage period. Similarly, total soluble solids and total sugars decreased with the increase in the storage period for both cultivars. Total anthocyanin content of both varieties remained almost constant throughout the storage period. Titratable acidity in Camarosa reduced with the increase in the storage period whereas it remained almost constant in Chandler. In case of ascorbic acid there was an increase in case of Camarosa whereas the same decreased significantly in Chandler. Significantly higher contents of esters and terpenoids in Camarosa indicated a better retention of the typical fruits flavour of strawberry compared to that of Chandler.

## Figures and Tables

**Table 1:** Identified aroma compounds by SPME method in both cultivars of strawberry stored at 5°C.

Compound	Relative concentration of aroma volatiles (%)							
	Camarosa				Chandler			
	Days of storage				Days of storage			
	0	3	6	9	0	3	6	9
Esters								
Methyl butanoate	0.070	0.079	0.079	0.089	0.039	0.036	0.034	0.031
Ethyl butanoate	0.060	0.159	0.256	0.327	0.075	0.098	0.289	0.292
Hexyl butanoate	0.350	0.328	0.292	0.246	0.270	0.301	0.376	0.375
Methyl hexanoate	9.360	8.276	5.432	4.488	0.007	0.189	0.400	0.505
Ethyl hexanoate	2.701	2.692	2.802	3.658	0.024	0.021	0.179	0.272
Hexyl hexanoate	0.157	0.181	0.196	0.162	1.050	1.053	0.848	0.898
Propyl hexanoate	0.222	0.202	0.238	0.246	1.042	0.876	0.645	nd
Methyl octanoate	0.232	0.243	0.230	0.247	0.033	1.761	5.238	8.254
Benzyl acetate	0.380	0.321	0.142	0.061	0.055	0.796	0.621	0.898
n-Octyl acetate	0.926	0.869	0.421	0.559	0.100	0.098	0.231	0.243
n-Octyl butyrate	0.480	0.431	0.334	0.392	1.143	1.013	0.901	0.915
(2Z)-2-Hexenyl butyrate	0.410	0.376	0.319	0.208	0.190	0.178	0.191	0.170
Terpenoids								
Linalool	0.611	0.602	0.592	0.569	0.426	0.536	0.839	1.704
*α*-Terpineol	0.197	0.182	0.170	0.132	nd	nd	nd	nd
*α*-Terpinene	0.024	0.029	0.041	0.062	0.029	0.023	0.017	0.025
Nerolidol	19.836	19.129	17.281	11.717	1.653	1.532	0.989	1.173
Alcohols and aldehydes								
1-Nonanol	0.403	0.482	0.768	1.050	0.029	0.018	0.023	0.187
2-Hexen-1-ol, (z)	2.006	2.004	2.121	2.836	1.042	1.041	1.079	1.488
2-Hexenal, (E)	0.031	0.025	0.032	0.036	0.021	0.019	0.017	0.020
2-Hexyl-1-octanol	0.145	0.018	0.075	0.082	0.170	0.009	nd	nd
Furanones								
2,5-Dimethyl-4-hydroxy-3(2H)-furanone	0.278	0.243	0.412	0.267	0.013	0.013	0.06	nd
Mesifurane	0.232	0.232	0.208	0.222	nd	nd	nd	nd
Others								
*α*-Farnesene	0.470	0.462	0.436	0.530	0.018	0.111	0.271	0.363
Dodecalactone	0.530	0.531	0.438	0.506	nd	nd	nd	nd
